# The Effects of Vinegar Processing on the Changes in the Physical Properties of Frankincense Related to the Absorption of the Main Boswellic Acids

**DOI:** 10.3390/molecules24193453

**Published:** 2019-09-23

**Authors:** Dongrui Liang, Zhangchi Ning, Zhiqian Song, Chun Wang, Yuanyan Liu, Xiaoying Wan, Shitao Peng, Zhenli Liu, Aiping Lu

**Affiliations:** 1 Institute of Basic Theory for Chinese Medicine, China Academy of Chinese Medical Sciences, Beijing 100700, China; ldr0310@163.com (D.L.); yizhangyichi1573@sina.com (Z.N.);; 2 School of Chinese Materia Medica, Beijing University of Chinese Medicine, Beijing 100029, China; 3 School of Chinese Medicine, Hong Kong Baptist University, Hong Kong 00825, China

**Keywords:** frankincense, vinegar-processed, changes in the physical properties, intestinal absorption effects

## Abstract

Boswellic acids (BAs), as the main components of frankincense, exhibit notable anti-inflammatory properties. However, their pharmaceutical development has been severely limited by their poor oral bioavailability. Traditional Chinese medicinal processing, called Pao Zhi, is believed to improve bioavailability, yet the mechanism is still completely unclear. Previous research suggested that the bioavailability of a drug can be influenced by physical properties. This paper was designed to investigate the physical properties of frankincense and processed frankincense, including the surface morphology, particle size, polydispersity index (PDI), zeta potential (ZP), specific surface area, porosity, and viscosity. The differences in the intestinal absorption characteristics and equilibrium solubilities between frankincense and processed frankincense were determined by an ultra-high-performance liquid chromatography coupled with a triple quadrupole electrospray tandem mass spectrometry (UHPLC-TQ-MS) analysis method. The results showed that vinegar processing can alter the surface morphology, decrease the particle size and PDI, raise the absolute values of the ZP, specific surface area and porosity, and drop the viscosity of frankincense. Meanwhile, the rates of absorption and dissolution of the main BAs were increased after the processing of frankincense. The present study proves that the physical properties were changed after processing, in which case the bioavailability of frankincense was enhanced.

## 1. Introduction

Frankincense is the oleo gum-resin obtained from trees of the genus Boswellia (family Burseraceae) native to the Arabian Peninsula (*Boswellia sacra*), Africa (*Boswellia carteri*, *Boswellia frereana*), and India (*Boswellia serrata*) [1,2]. As an essential drug in traditional Chinese medicine (TCM), frankincense is widely used for the treatment of inflammatory diseases such as rheumatism, rheumatoid arthritis [3], and osteoarthritis [4,5]. The main constituents of frankincense are monoterpenes, diterpenes, lipophilic pentacyclic triterpene acids, polysaccharides, and volatile oil [6]. The presence of pentacyclic triterpene acids, such 11-keto-β-boswellic acid (KBA), 3-acetyl-11-keto-β-boswellic acid (AKBA), α-boswellic acid (α-BA), β-boswellic acid (β-BA), 3-acetyl-α-boswellic acid (α-ABA), and 3-acetyl-β-boswellic acid (β-ABA) was proven to mainly contribute to the anti-inflammatory activity [7,8].

However, the main boswellic acids (BAs) of frankincense showed low bioavailability, which has been a challenge in the successful formulation of therapeutic products [9]. The low brain availability in rats and serum levels in humans of six major BAs were determined [10]. Buchele B et al. analyzed 12 different pentacyclic triterpenic acids in human plasma [11], which have demonstrated the low bioavailability of BAs, particularly KBA and AKBA, in animals and human. Nowadays, many bio-derivatization and chemical modification approaches have been performed to make better use of different BAs, such as applying new formula compatibilities or administering them with anionic drugs and standardized meals [9]. However, the results of previous research are still not satisfactory.

Chinese medicinal processing, called Pao Zhi, is a pharmaceutical technique for different therapeutic uses. According to TCM theory, various processing procedures, such as stir-frying, soaking with water, heating with vinegar, or steaming with alcohol, were used on most Chinese medicinal herbs [12]. In recent decades, appropriate processing approaches have been used to reduce the toxicity and exert a large maximal therapeutic efficacy, such as vinegar stir-frying and wine stir-frying [13]. Moreover, their effects on improving the bioavailability cannot be ignored either. Results indicate that the salt-processing of scurfpea fruit can significantly increase the distribution of psoralen and isopsoralen to generative organs [14]. Wine processing was proven to exert effects on the absorptions of most of the flavonoids, according to the the results of a comparative pharmacokinetic study of crude and wine-processed *Radix Scutellariae* [15]. Similarly, vinegar processing altered the *AUC* and *C_max_* parameters of the saikosaponin b1, b2, a, c, and d in *Bupleuri Radix* (BR, Chaihu in Chinese) [13]. Treatment with the processed frankincense significantly increased the levels of *AUC*, *C_max_*, *T_1/2_* and *MRT* of KBA and AKBA in rats compared with the crude sample [12]. However, the mechanism of the bioavailability enhancement effect of processing is still completely unclear.

Drugs with low oral bioavailability have been an ongoing challenge in the successful formulation of therapeutic products [16]. Bioavailability is influenced by the formulation, physicochemical properties, excipients, and preparation technology of drugs [17]. It has been proven that when the surface area becomes larger, the dissolution rate of the drug is promoted, and thus, the bioavailability is improved [18,19]. Another physical factor responsible for poor bioavailability is the viscosity. In addition, a decrease in viscosity leads to an increase in the rates of drug dissolution and diffusion and tends to enhance the absorption [20,21]. Reducing the agglomeration of powders is also generally regarded as an important factor for increasing the particle size and surface area, which can promote the absorption of the drug [22].

Moreover, the solubility, stability, dissolution rate, and absorption of drugs in the gastrointestinal tract are the key parameters affecting the bioavailability [23,24]. Recently, a variety of strategies have been explored to overcome the low oral bioavailability of Chinese medicinal herbs [25,26,27]. However, little is known about the enhancement of the bioavailability of the drug after processing and changing the physical properties.

In this paper, the differences between the physical properties of frankincense and processed frankincense were compared by evaluating various parameters, including the surface morphology, particle size, polydispersity index (PDI), zeta potential (ZP), specific surface area, porosity, and viscosity. The absorption characteristics in rat intestinal segments and the differences in the equilibrium solubility (pH = 2.0, 7.0, 7.8–8.0) between frankincense and processed frankincense were observed as well. This study is helpful to define and understand the mechanism for the bioavailability enhancement after vinegar processing.

## 2. Results and Discussion

### 2.1. Differences in Physical Property Indices Between Frankincense and Processed Frankincense

#### 2.1.1. Surface Morphology by Scanning Electron Microscopy (SEM)

**Figure 1:**
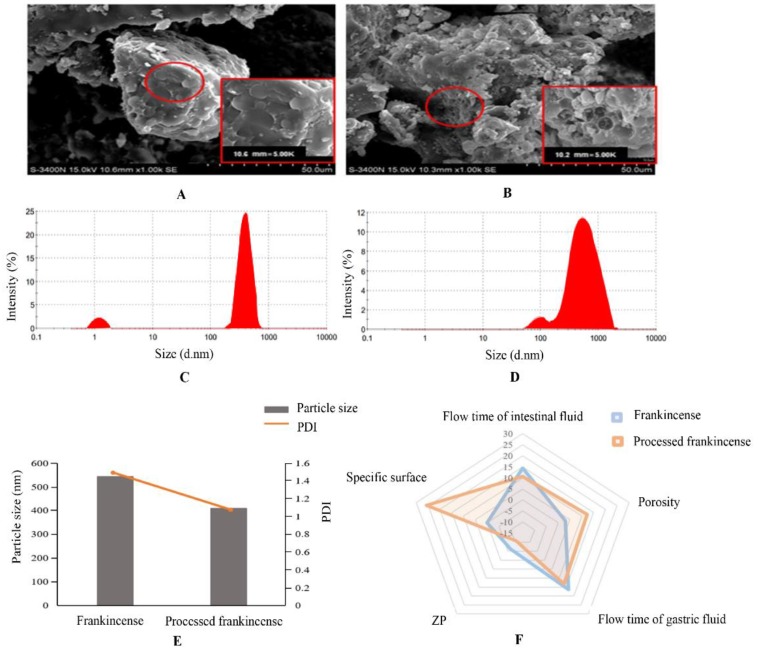
Physical property indices of frankincense and processed frankincense: (**A**) SEM image of frankincense; (**B**) SEM image of processed frankincense; (**C**) particle size distribution of frankincense; (**D**) particle size distribution of processed frankincense; (**E**) particle size and PDI of frankincense and processed frankincense; and (**F**) radar plots of five physical property indices (ZP, specific surface, porosity and flow times of intestinal and gastric fluid).

The absorption and metabolism of drugs in vivo are extremely complicated processes. The pharmacological effects depend not only on the chemical compositions or structures of the drug but also on its physical properties [21]. This measurement aimed to compare the surface morphologies of frankincense and processed frankincense. SEM has become an indispensable tool and was used for topography imaging of powder samples in this study [28,29]. SEM images of frankincense and processed frankincense exhibited a significantly different status of the powder sample (Figure 1A,B). The results showed the images of frankincense with a smooth surface, while the processed frankincense exhibited folds full of holes with a rough surface. Additionally, porous particles have a larger surface area than non-porous particles [30]. Thus, processed frankincense was believed to lead to an increase in the surface area. According to the Noyes–Whitney equation (Noyes and Whitney, 1897), the surface area determined the dissolution rate [18]. Furthermore, a high dissolution rate can promote drug absorption [19]. There are many Chinese traditional patent medicines containing vinegar-processed frankincense as a main ingredient smashed medicine for use, such as Qili san and Shixiangzhitong wan [31]. Therefore, it is possible to enhance the absorption performance and bioavailability by increasing the surface area of the powder after the processing of frankincense.

#### 2.1.2. Particle Size, PDI, and ZP

The particle size, PDI, and ZP are the most important characteristics of drugs. The following characteristics of drugs are closely related to the particle size: 1) drug saturation solubility, 2) physical stability, 3) dissolution rate, and 4) bioavailability [32]. The physical stability of drug solutions can also be indicated by PDI and ZP values [33,34]. In this work, particle size, PDI and ZP measurements are used to compare the frankincense and processed frankincense. The average particle size of processed frankincense was 409.300 ± 0.002 nm (Figure 1C–E) with a narrow size distribution and a PDI = 0.987 (Figure 1E). Meanwhile, the particle size of frankincense was 543.833 ± 0.071 nm (Figure 1C–E) with a PDI = 3.489 (Figure 1E). The results all suggested the statistically extreme significant differences between frankincense and processed frankincense (*p* < 0.01, *p* < 0.05). Vinegar processing mainly contributes to the crushing process of frankincense, thereby resulting in the decrease of the particle size. As clearly observed in Figure 1F, the ZP values of frankincense and processed frankincense were measured as −6.500 mV and −10.643 mV respectively (*p* < 0.01). In general, the low values of particle size, PDI, and high absolute values of the ZP are preferably desired for achieving a good stability [32,35]. On the other hand, a decrease in the particle size leads to an increase in the particle dissolution rate by the modified Noyes–Whitney law (Noyes and Whitney, 1897) [36]. A reduction in the particle size can result in an increase of the saturation solubility based on the Ostwald–Freundlich equation [37]. Furthermore, a high dissolution rate and solubility of the drug can improve its absorption performance and bioavailability [38]. As the results showed, it is not hard to observe that the values of the particle size and PDI decreased and the absolute values of the ZP increased, respectively, after stir-baking with vinegar. Therefore, the high stability and bioavailability of frankincense were anticipated from its lower particle size and PDI as well as higher ZP value obtained after processing.

#### 2.1.3. Specific Surface Area and Porosity

The specific surface area is defined by the International Union of Pure and Applied Chemistry (IUPAC). When the area of the interface between two phases is proportional to the mass of one of the phases, such as a solid adsorbent, an aerosol or an emulsion, the specific surface area is defined as the surface area divided by the mass of the relevant phase [39]. To detect the specific surface area of a particle, usually the inner and outer surface areas of a given sample are measured. Dividing this by the mass of the measured sample gives the specific surface area in m^2^/g [39]. Additionally, the porosity is defined as a parameter that has a great influence on the processing properties of the powder and the quality of its preparation [40]. As is shown in Figure 1 F, the surface area increased from 0.054 ± 0.01 to 25.738 ± 0.494 m^2^/g, while the porosity increased from 3.142 ± 0.054 to 12.348 ± 0.314% after the processing of frankincense. The results all showed the statistically extreme significant differences between frankincense and processed frankincense (*p* < 0.01). Vinegar processing mainly contributes to the crushing process of frankincense, thereby resulting in an increase of the specific surface area. The specific surface area can contribute to the improvement of drug solubility and dissolution behavior because a higher surface area could give rise to a higher dissolution rate [37]. Meanwhile, the high porosity also makes a contribution to dissolution enhancement and the consequently increased bioavailability of the drug [37,41]. The results indicated that the value of the specific surface area of the frankincense was increased and the porosity was decreased after processing. Therefore, all of these may improve the bioavailability of the BAs in the powder of processed frankincense compared to that in frankincense.

#### 2.1.4. Viscosity of the Simulated Gastrointestinal Fluid

The biopharmaceutical properties as well as physicochemical properties of the drug and its inter-relationship with the gastrointestinal tract contribute to the influence of the oral drug absorption process. The drug dissolution, solubility, and permeability across gastric and intestinal barriers are the key parameters controlling absorption [17]. Viscosity is an important physical and chemical feature. It is defined as the ability of the fluid to resist the flow [42]. This paper determined the difference in viscosity between frankincense and processed frankincense by measuring the flow time of simulated gastric fluid and intestinal fluid. As observed in Figure 1F, the flow times of simulated gastric fluid and intestinal fluid of processed frankincense were 13.23 ± 0.28 and 10.66 ± 0.49 min, while those of frankincense were 16.47 ± 0.24 and 14.48 ± 0.22 min. The results all indicated statistically significant differences between frankincense and processed frankincense (*p* < 0.05). A decrease in viscosity leads to increases in the rates of drug dissolution and diffusion [20]. In addition, the viscosity tends to affect the absorption [21]. According to the results of the flow time, that of the processed sample is shortened, which indicated a decrease in viscosity after processing. Thus, processing will increase the rates of drug dissolution and diffusion as well as improve the drug absorption [20,43].

### 2.2. Establishment of the Content Determination Method of the Six Main BAs

#### 2.2.1. Optimization of Sample Preparation

To optimize and validate our previous extraction protocol for frankincense, processed frankincense and intestinal absorption solution, we optimized the different extraction methods for frankincense and processed frankincense, such as ethanol extraction, water extraction, and steam distillation-ethanol continuous extraction. The optimum ethanol extraction process was as follows: 100 g of sample was extracted with 30 times the amount of ethanol and sonicated for 30 min. In the same extraction conditions, ethanol extraction is superior to the other extraction methods. Moreover, compared to traditional extraction methods, the ultrasonic method requires no heating. Therefore, the ultrasonic method could avoid the influence of heating and improve the extraction rates for frankincense and processed frankincense.

We also optimized the extraction solvent, evaluating chloroform, ether, and ethyl acetate for the intestinal absorption solution. The results showed that ethyl acetate could be applied for an excellent extraction rate and good separation effect for intestinal absorption.

#### 2.2.2. Optimization of the LC Conditions

To achieve efficient and rapid analysis, different mobile phases (including methanol–water, acetonitrile–water, methanol–formic acid solution, and acetonitrile–formic acid solution), column temperatures (25, 30, and 35 °C), mobile phase compositions (MeOH-H_2_O, MeCN-H_2_O, MeOH-H_2_O (containing 0.2% formic acid and 5mM ammonium formate)) and flow rates (0.4, 0.5, and 0.6 mL/min) were examined and compared. Because of the limit of mass spectrometry in the separation of isomers, the isomers such as α-BA/β-BA and α-ABA/β-ABA can just be separated by the chromatography behavior. As a result, excellent separation was achieved in the shortest analysis time when methanol-water was used at a flow rate of 0.5 mL/min with a column temperature of 30 °C. The addition of 0.2% formic acid and 5 mM ammonium formate was proved to improve the sensitivity and the peak shape obviously.

#### 2.2.3. Optimization of the MS Conditions

**Figure 2:**
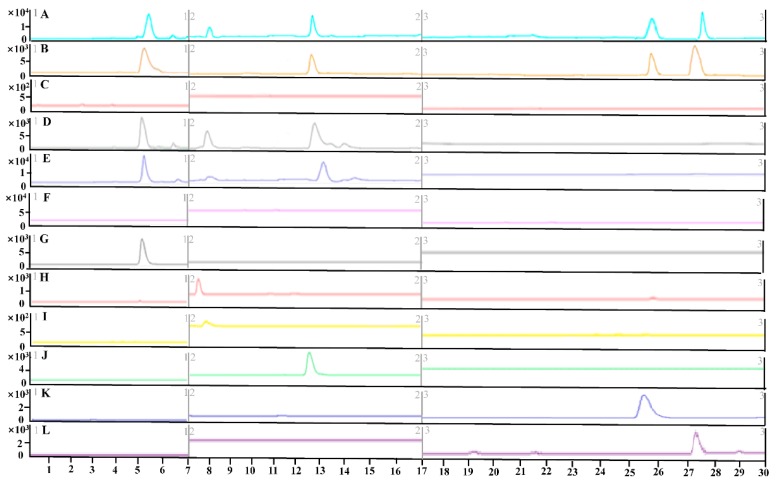
UHPLC-TQ-MS chromatography of an ethanol solution, ethanol extracts of frankincense and processed frankincense, frankincense solution, processed frankincense solution, and blank Tyrode′s solution of the intestine sack, as well as standard solutions: (**A**) ethanol extracts of processed frankincense; (**B**) ethanol extracts of frankincense; (**C**) ethanol solution; (**D**) processed frankincense solution of the intestine sack; (**E**) frankincense solution of the intestine sack; (**F**) blank Tyrode′s solution of the intestine sack; (**G**) KBA; (**H**) AKBA; (**I**) α-BA; (**J**) β-BA; (**K**) α-ABA; and (**L**) β-ABA.

The MS/MS fragmentation patterns were investigated to develop an accurate and sensitive quantitative method. All factors related to MS performance have been optimized, including the ionization mode, capillary voltage, fragmentor voltage, collision energy, gas flow, and desolvation temperature. MS spectra were investigated in both the positive and negative modes at first. The results showed maximum sensitivity when operating in the negative ion mode. Moreover, multiple reaction monitoring (MRM) was performed to increase the specificity and sensitivity of quantification. The retention time (RT) and MS information for each component including [M + H]^+^, daughter ion, fragmentor, and CE are shown in Table 1. Meanwhile, the ultra-high-performance liquid chromatography coupled with a triple quadrupole electrospray tandem mass spectrometry (UHPLC-TQ-MS) chromatography of frankincense, processed frankincense and blank Tyrode′s solution of the intestine sack as well as the standards are shown in Figure 2. The results indicated that all compounds were specific to their corresponding MRM transitions.

### 2.3. Method Validation

#### 2.3.1. Specificity

Under the conditions described above, the UHPLC-TQ-MS chromatography of ethanol solution, ethanol extracts of frankincense and processed frankincense, frankincense solution, processed frankincense solution, and blank Tyrode′s solution of the intestine sack, as well as standard solutions of KBA, AKBA, α-BA, β-BA, α-ABA, and β-ABA are shown in Figure 2. No significant interferences were observed during the UHPLC-TQ-MS chromatography of analytes, suggesting the acceptable specificity of the proposed method.

#### 2.3.2. Linearity, Limit of Detection (LOD), and Lower Limit of Quantitation (LLOQ)

The detailed information regarding calibration curves, linear ranges, LOD and LLOQ is presented in Table 2. The regression equations of KBA, AKBA, α-BA, β-BA, α-ABA, and β-ABA were Y = 2145.1X − 25.865, Y = 19549X − 151.53, Y = 1169.3X − 7.466, Y = 170852X + 4894.1, Y = 300.64X − 46.221, and Y = 98.805X − 29.213, respectively. The calibration curves exhibited good linearity (r > 0.9925) within the test range. The values of the LOD were 0.021, 0.014, 0.032, 0.011, 0.33, and 0.28 mg/mL, respectively. The values of the LLOQ were 0.054, 0.039, 0.087, 0.035, 0.79, and 0.59 mg/mL, respectively. The LOD ranged from 0.011 to 0.33 mg/mL, and the LLOQs ranged from 0.035 to 0.79 mg/mL, respectively. The values could meet the requirements of the determination of the six BAs.

#### 2.3.3. Precision and Accuracy

The results for the intra-day and inter-day precision and accuracy in samples are summarized in Table 3. The intra-day and inter-day precision in the standard mixture ranged from 0.92 to 5.01%. The values were presented as relative standard deviation (RSD). The accuracy values presented as coefficient of variation (CV) were in the range from 0.59 to 3.85% for the extraction of frankincense and 0.64 to 54.66% for quality control (QC) samples at low, middle and high QC levels. According to the Food and Drug Administration (FDA) guidelines, the intra-day and inter-day precision and accuracy values measured at low, middle, and high concentration levels should not exceed 15%, whereas they should not exceed 20% for the LLOQ. The results revealed good precision and accuracy.

#### 2.3.4. Matrix Effect

Undetected substances that coelute with the analyte may influence the signal intensity corresponding to the mass transition of that analyte, thus affecting the linearity and precision of the method. This is an influence called the matrix effect. The matrix effects at the low quality control (LQC), middle quality control (MQC) and high quality control (HQC) levels were (94.6 ± 1.7)%, (92.9 ± 5.2)%, and (97.0 ± 4.1)% for KBA; (98.2 ± 4.5)%, (97.3 ± 1.1)%, and (95.5 ± 2.7)% for AKBA; and (96.3 ± 2.5)%, (98.6 ± 3.1)%, and (97.7 ± 4.6)% for α-BA. The matrix effects of β-BA, α-ABA and β-ABA were (96.8 ± 2.5)%, (94.1 ± 1.6) %, and (98.9 ± 2.1)%; (95.0 ± 3.7)%, (94.3 ± 1.9)%, and (95.8 ± 2.0)%; and (97.1 ± 3.4)%, (93.4 ± 2.2)% and (96.9 ± 4.0)% at the LQC, MQC, and HQC levels, respectively These results showed no obvious influence of the matrix effect for each analyte at three concentration levels (low, middle, and high).

#### 2.3.5. Recovery

The recoveries of extraction samples of frankincense and processed frankincense varied between 96.71 and 101.01% with RSD less than 4.86%, and those of the QC samples varied between 91.68 and 106.44% with RSD less than 4.48%. The details for the recoveries are shown in Table 4. These results showed that the assay is a useful and reliable method for determining the contents of six analytes in extraction samples and QC samples at low, middle, and high levels.

#### 2.3.6. Stability

The stabilities of QC samples were evaluated at the low, medium and high levels under different storage conditions. The results for samples of QC samples as well as extracts of frankincense and processed frankincense are listed in Table 5. The RSD% were within 15% for all stability tests. The stabilities (RSD) of QC samples varied between 1.08% and 4.87%, and the stabilities of extraction samples were less than 4.39%. The results were considered to be satisfactory and within the acceptable limits, thus making the method applicable for routine analysis.

#### 2.3.7. Carryover

The chromatogram following the upper limit of quantification suggested no peak interference in an analytical run, near the RT of each analyte. The results showed that carryover met the acceptance criteria.

### 2.4. Determination of the Intestinal Absorption Rate

**Figure 3:**
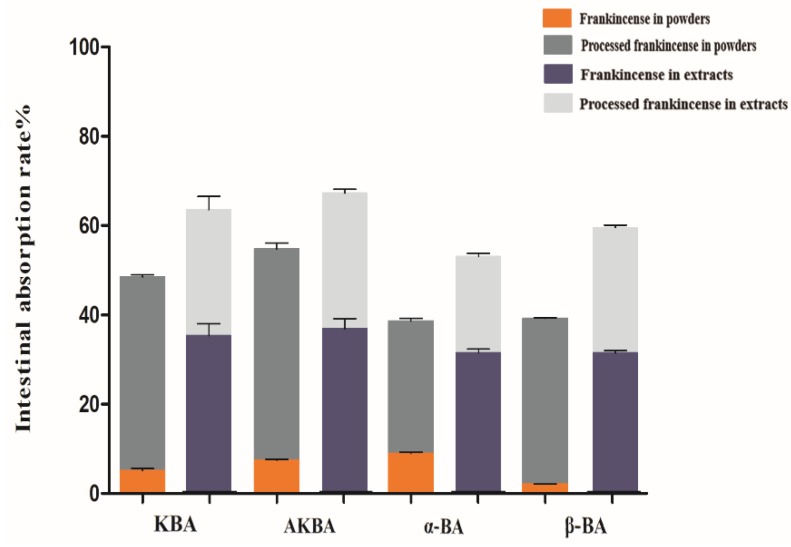
Intestinal absorption rates of frankincense and processed frankincense in powders and ethanol extracts.

Drugs are absorbed into the blood-stream mainly in the intestine [44]. The everted intestine sac was first described by Wilson and Wisemans and used to study drug absorption and metabolism [45,46]. Therefore, everted rat gut sacs were studied to determine the intestinal absorption rates of frankincense and processed frankincense both in powders and ethanol extracts. In the present study, we observed that there were differences in the absorptions of four BAs before and after processing. It can be seen from Figure 3 that the absorption rates of KBA, AKBA, α-BA, and β-BA in frankincense are 5.14 ± 0.88, 7.41 ± 0.42, 8.93.12 ± 0.41, and 2.06 ± 0.07 in powders and 20.37 ± 0.56, 28.89 ± 0.72, 31.21 ± 1.33, and 17.80 ± 0.66 in ethanol extracts. The absorption rates of processed frankincense are 48.47 ± 0.51, 54.72 ± 2.46, 30.64 ± 0.52, and 39.26 ± 0.22 in powders and 72.80 ± 0.78, 82.17 ± 4.12, 61.32 ± 1.29, and 69.10 ± 0.81 in ethanol extracts, respectively. The absorption rates of α-ABA and β-ABA were not detected. The findings demonstrate that the absorption rates of the main BAs in processed frankincense are higher than in frankincense (*p <* 0.01, *p <* 0.05). In addition, the intestinal absorption rates of frankincense and processed frankincense in powders are both lower than those in ethanol extracts (Figure 3). However, by comparing the difference values, the values of powders are higher than those of ethanol extracts (Figure 3), i.e., the powders of frankincense are believed to produce much greater change after processing. The difference is statistically significant (*p <* 0.01, *p* < 0.05). The explanation for this is that several physical properties of frankincense—such as the particle size, specific surface area, and so on—were changed after processing. Therefore, the increase in intestinal absorption rates reveals that the drug absorption and bioavailability can be improved after the processing of frankincense and the absorption rate is affected by the physical properties of frankincense and processed frankincense to the greatest degree.

### 2.5. Measurement of the Equilibrium Solubility

**Figure 4:**
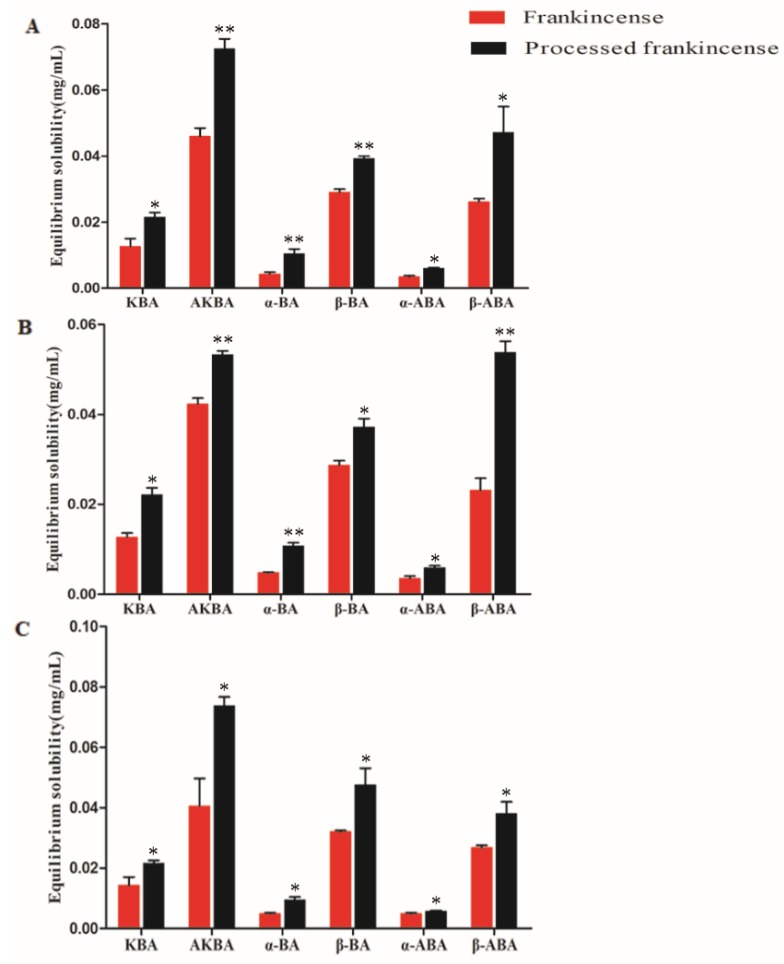
Equilibrium solubilities of six BAs in buffer solutions with different pH values: (**A**) pH = 2.0; (**B**) pH = 7.0; and (**C**) pH = 7.8–8.0. * indicates a statistically significant difference between frankincense and processed frankincense (*p* < 0.05). ** indicates a statistically highly significant difference between frankincense and processed frankincense (*p* < 0.01).

Equilibrium solubility is an important physicochemical parameter affecting the efficacy and absorption of drugs in the gastrointestinal tract [47]. A high equilibrium solubility contributes to the drug absorption performance and bioavailability [21]. Therefore, it is necessary to study the equilibrium solubility of frankincense and processed frankincense to address the differences in drug absorption between them. Dissolution of the sample is a critical part in the determination of the equilibrium solubility [48]. According to the methods for the measurement of equilibrium solubility in many studies, shaking for 24 h is a good method to dissolve and avoid degradation of the sample. Thus, the method of shaking for 24 h was applied in this paper. As shown in Figure 4, the equilibrium solubilities of processed frankincense in buffer solutions with different pH values are higher than those of frankincense, and the differences are all significant between them. The results suggested that the drug absorption and bioavailability could be increased after the processing of frankincense.

## 3. Materials and Methods

### 3.1. Instruments, Chemicals and Animals

The Ostwald-type viscosimeter was supplied by the Malvern Co., Ltd. (Malvern, UK). The HITACHI E-1010 ion sputtering instrument and SEM were procured by the Hitachi Co., Ltd. (Tokyo, Japan). An Auto Pore IV 9500 mercury porosimeter was purchased from the Micromeritics Co., Ltd. (Atlanta, GA, USA). The Zetasizer Nano ZS980 laser particle size analyzer (LPSA) was provided by the Malvern Co., Ltd. (Malvern, Worcestershire, UK). The Empower 2 data processing system was obtained from the Waters Co., Ltd. (Milford, MA, USA). A HSS-1 B digital thermostat bath from the Chengdu Instruments Factory (Chengdu, Sichuan, China) and a SorvallsuperT21 high-speed centrifuge from the DuPont Co., Ltd. (Wilmington, DE, USA) were used. The AL-F_2_11AE ASD electromagnetic furnace was provided by the ASD Electric Appliance Co., Ltd. (Taizhou, Zhejiang, China). The 1260 high performance liquid chromatograph (HPLC, G1322A degasser, G1311A quaternary pump, G1313A automatic sampler, G1316A incubator, and HP chem-station) and 6410ATQ mass spectrometer were supplied by the Agilent Co., Ltd. (Santa Clara, CA, USA). The TCQ-250 ultrasonic cleaner was from the Beijing Medical Equipment Factory (Beijing, China). A CP225D 1/10 million electronic balance was obtained from the Sartorius Co., Ltd. (Göttingen, Germany). The Titramax 100 oscillator was from the Heidolph Co., Ltd. (Schwabach, Germany).

The standards of KBA and AKBA were from the National Institutes for Food and Drug Control of China (Beijing, China). The α-BA, β-BA, α-ABA, and β-ABA were from the ChromaDex Co., Ltd. (Irvine, CA, USA). LongMen rice vinegar was obtained from the Beijing Ershang Longhe Food Co., Ltd. (Beijing, China). Sodium carboxymethyl cellulose (C_8_H_16_NaO_8_, CMC-Na) was obtained from the Sinopharm Chemical Reagent Co., Ltd. (Shanghai, China). Analytical-grade methanol, ethanol, and ethyl acetate produced by the Beijing Factory of Chemical Technology (Beijing, China) were used. Other analytical-grade reagents, such as formic acid, ammonium formate, acetic acid, acetonitrile, phosphoric acid, sodium dihydrogen phosphate, magnesium chloride, potassium chloride, sodium hydrogen carbonate, sodium chloride, and glucose were supplied by the Sinopharm Chemical Reagent Co., Ltd. (Shanghai, China). Five batches of frankincense were procured from the Beijing Tongrentang Co., Ltd. (Beijing, China) and the plant species were identified to belong to *Boswellia papyrifera* according to the literature [9]. The processed sample was prepared in the lab using the established method [49]. Briefly, 200 g frankincense was put into a heated pot stir-fried quickly and sprayed rice vinegar until the surface is glossy. The frankincense and processed frankincense were crushed under the same conditions and passed through a 100~120 mesh sieve to obtain their powder samples.

Male Wister rats aged 10 weeks with a mean weight of 280–300 g were purchased from the Research Institute of Experimental Animals, Chinese Academy of Medical Science. The rats were fed with food and water ad libitum and then were allowed to acclimatize themselves for 1 week before the initiation of the experiment. The rats were housed in a temperature of 20–26 °C, humidity of 40–70%, and light-controlled environment. The light–dark cycle was 12 h, with the light phase being set from 6:00 am. to 6:00 pm. The rodent license of the laboratory (no. SYXK 11-00-0039) was issued by the National Science and Technology Ministry of China. This study was approved by the Research Ethics Committee of the Institute of Basic Theory of Chinese Medicine, China Academy of Chinese Medical Sciences.

### 3.2. Measurement of the Physical Property Indices of Frankincense and Processed Frankincense

#### 3.2.1. Measurement of the Surface Morphology

One gram of powdered sample was sputter-coated with gold under vacuum, and then, the surface morphology was evaluated by SEM.

#### 3.2.2. Measurement of the Particle Size, PDI, and ZP

A mixture of 1.0 g of powdered sample and 10.0 mL distilled water was sonicated at 20 ± 2 °C and 250 W for 20 min in a conical flask with a cover. The samples were stored at 4 °C and mixed before use. The particle size, PDI, and ZP of the obtained solution were measured by a laser particle size analyzer. Each experiment was repeated three times, and the presented data represent the means ± SD.

#### 3.2.3. Measurement of the Specific Surface Area and Porosity

The specific surface area and porosity of samples were determined by a mercury intrusion porosimeter (AutoPore IV 9500, Micromeritics, Norcross, GA, USA) with the pressure ranging from 0.10 psi to 60000.00 psi. Before the determination, the low-pressure, pressure, and high-pressure parameter ranges were set from 10 kPa to 228 kPa, 0 kPa to 228 kPa, and 0.1 MPa to 228.0 MPa, respectively. Each experiment was repeated three times, and the presented data represent the means ± SD.

#### 3.2.4. Measurement of the Viscosity [31]

One gram of powdered sample was dispersed in 10.0 mL of simulated gastric fluid or intestinal fluid, respectively. The Ostwald-type viscosimeter was used to measure the flow time, and each experiment was repeated three times. The presented data represent the means ± SD. The accuracy of measurement was evaluated by the time required for a given volume of the reference liquid. The precision of the measurement was evaluated by the SD value of repeated trials.

### 3.3. UHPLC-TQ-MS Conditions

The UHPLC-TQ-MS analyses were carried out on a reversed-phase Agilent XDB C_18_ column (4.6 × 100 mm, 1.8 μm) (Agilent, Santa Clara, USA). The mobile phase consisted of (A) water (containing 0.2% formic acid and 5 mM ammonium formate) and (B) methanol. The flow rate was 0.5 mL/min. The column temperature was 30 °C. The gradient program was used for rapid separation as follows: 85–85% B (0–20 min), 85–98% B (20–35 min), and 98%–98% B (35–40 min). The wavelengths were 210 nm, 250 nm, and 280 nm. The conditions of MS analysis were as follows: drying gas temperature, 300 °C, gas flow rate, 10 L/min, and nebulizer pressure, 45 psi. ESI in the negative ionization mode was used, and the capillary voltage was set to 4000 V. The MRM mode was employed to quantify the six BAs.

### 3.4. Preparation of Standard Solutions

Certain amounts of KBA, AKBA, α-BA, β-BA, α-ABA, and β-ABA were dissolved in methanol, respectively, to obtain standard stock solutions, and they were stored below 4 °C.

The mixed standard solutions of KBA, AKBA, α-BA, β-BA, α-ABA, and β-ABA at concentrations of 1.04, 1.02, 1.06, 1.01, 0.98, and 0.98 mg/mL respectively were obtained by dilution of the stock solution in different solvents. For the quantitative assay of the frankincense extraction assay, the serial dilutions of mixed standard solutions were prepared using methanol. For the intestinal absorption experiment, the serial dilutions were performed using blank Tyrode′s solution of the intestine sack.

### 3.5. Preparation of QC Samples

An equal volume of intestinal absorption solution in all sac groups was collected. To improve the reliability of statistical data and correct for signal drift during sample runs, the mixed standard solution was spiked in the homogenized biological matrices of intestinal absorption solution to obtain QC samples. QC samples were prepared at low, middle, and high concentration levels for use in the further method validation experiments.

### 3.6. Method Validation

#### 3.6.1. Specificity

For the specificity assessment, ethanol solution, ethanol extracts of frankincense and processed frankincense, frankincense solution, processed frankincense solution, and blank Tyrode′s solution of the intestine sack as well as standard solutions were examined by the proposed method to test for endogenous interference around the RT of the components. The peak areas of the endogenous components which co-eluted with the compound of interest should be less than 20% of the peak area of the LLOQ.

#### 3.6.2. Linearity, LOD, and LLOQ

The quantitative analysis used an external calibration method, and the linear calibration curves were constructed by six different concentrations of the mixed solution of standard samples. Each concentration was analyzed in triplicate, and the calibration curves were established by plotting the peak areas versus the concentrations of each solution. The LOD and LLOQ were measured at signal-to-noise ratios (S/N) of 3 and 10, respectively, as per criteria from [50].

#### 3.6.3. Precision and Accuracy

The intra-day precisions for samples were evaluated by measuring the mixed solution of standard samples in low, middle, and high concentration levels six times a day, while inter-day precisions were assessed twice a day on three consecutive days. The intra-day or inter-day precision was expressed as the RSD.The accuracy was assessed using the extracts of frankincense as well as QC samples. It was calculated on the basis of the given formula (mean concentration found/concentration taken) × 100. The accuracy was expressed as the CV.

#### 3.6.4. Matrix Effect

The matrix effect was determined by comparing the peak area ratios of analytes in the spiked post-extraction QC samples (A) with the peak area ratios of neat standard solutions (B) at the equivalent concentration with three replicates each at LQC, MQC, and HQC levels.

#### 3.6.5. Recovery

The standard addition method was conducted to measure the analyte recoveries in the extracts of frankincense and processed frankincense as well as in QC samples.

The recoveries for biological samples were performed by comparing the peak areas of QC samples with the peak area of spiked QC samples (known amounts of mixed standard solution spiked in QC samples).

Known amounts of mixed standard solution were added to known amounts of six independent solutions including the extracts of frankincense and processed frankincense.

#### 3.6.6. Stability

Stability tests were performed to measure the analyte stability in the extracts of frankincense and processed frankincense as well as in QC samples under different conditions.

As for the stabilities of the extracts of frankincense and processed frankincense, the extraction solutions of samples were analyzed at 0, 2, 4, 6, 8, 10, 12, and 24 h with three replicates each at low, middle, and high level. The analytes were considered to be stable for 24 h when the RSD was within 5% of the theoretical concentration.

For QC samples, the bench top stability (25 °C, 6 h), autosampler stability (4 °C, 9 h), freeze-thaw stability (−80 °C, 12–16 h) and long-term stability (4 °C, 30 days) were measured with three replicates each at LQC, MQC, and HQC levels. QC samples were considered to be stable when values of the assay were within the acceptable limits of precision (≤15% RSD) and accuracy (± 15% SD).

#### 3.6.7. Carryover

Carryover may be reflected in subsequent runs. As a result, a test was conducted to verify any carryover of analytes. Carryover was assessed by injecting the biological samples at the highest standard concentration of the calibration curve followed by the lowest standard concentration and a series of blank injections. The carryover was considered to be acceptable when the carryover was at 20% corresponding to the peak area of the LLOQ level according to the EMA guidelines for bioanalysis.

### 3.7. Procedure of Intestinal Absorption

#### 3.7.1. Preparation of Frankincense and Processed Frankincense Solutions

CMC-Na was diluted with Tyrode′s solution (in g/L, 8.0 NaCl, 0.28 KCl, 0.05 NaH_2_PO_4_, 1.0 NaHCO_3_, 0.1 MgCl_2_, 1 H_2_O, 0.2 CaCl_2_, and 1.0 glucose) to a concentration of 1%.

Three grams of frankincense or processed frankincense powder was dispersed separately in 500 mL of CMC-Na (1%) to obtain a suspension of the powder sample.

The frankincense (100 g) and processed frankincense powders (100 g) were extracted with 3 L of ethanol under sonication for 30 min. After filtration and evaporation, the ethanol extracts of frankincense (69.38 g) and processed frankincense (54.05 g) were obtained with yields of 69.38% and 54.05%. frankincense extract (2.08 g) and 1.62 g of the processed frankincense extract were dispersed separately in 10 mL of ethanol. Then, the above ethanol solutions of frankincense and processed frankincense were dissolved in 500 mL of CMC-Na (1%) by vortex mixing to obtain the sample solution of ethanol extracts.

#### 3.7.2. Preparation of the Everted Rat Gut Sacs and Intestinal Absorption Solution

All of the rats were starved for 12 h, and standard water was provided before the experiments. The rats were sacrificed by decapitation. After a laparotomy, 11 cm of the upper end of the duodenum, jejunum, ileum, and colon was excised and washed with Tyrode′s solution at 0 °C.The clean intestinal tract was prepared into sacs with 2 mL of Tyrode′s solution in the blank group by ligation via a blunt needle and the other end tied. Each sac was placed in a bath that contained 25 mL of Tyrode′s solution maintained at 37 °C and constantly gassed with O_2_ for 5 min (95% O_2_/5% CO_2_). Then, 25 mL of the sample solution of ethanol extract and CMC-Na suspension of powdered sample were prepared in the bath. Two hours later, the intestinal absorption solution in the sacs was collected to accurately calculate the volume, and each sac was accurately weighed before and after being cut open. All of the samples were stored at −20 °C.

#### 3.7.3. Sample Preparation

Three milliliters of the above intestinal absorption solution was placed into a 50 mL polyethylene centrifugal tube, then, 10 mL of ethyl acetate was added and vortex mixed. The process was repeated three times. After that, the supernatants were combined and reduced to dryness in a gentle nitrogen stream, and added into 100 μL of methanol for dissolution. The mixture was filtered into HPLC vials through a Millipore filter (0.22 µm).

#### 3.7.4. Measurement of the Rate of Intestinal Absorption

All solutions of the four intestinal segments were analyzed using the UHPLC-TQ-MS method. After analysis, the amounts (ng/mm^2^) of the six main BAs components of frankincense and processed frankincense and the rate of intestinal absorption were calculated. The presented data represent the means ± SD.

### 3.8. Measurement of the Equilibrium Solubility

#### 3.8.1. Preparation of Samples

Accurately weighed powder (2 g) samples of frankincense and processed frankincense were dispersed in 10 mL of distilled water, phosphate buffer solution (PBS) at a pH of 2.0 or PBS at a pH of 7.8~8.0 separately and then sonicated at 20 ± 2 °C and 250 W for 30 min in 50 mL conical flasks with covers. The flasks were continuously shaken horizontally at (37 ± 1) °C and (100 ± 1) r/min for 24 h (Heidolph Titramax 100, Schwabach, Germany) until the equilibrium plateau was reached to obtain the solution.

Five milliliters of the above solution was extracted three times with the ethyl acetate (3 × 10 mL) to ensure complete extraction. All three extracts were pooled and concentrated in a rotary evaporator under vacuum to be completely dried and dissolved in 5 mL of methanol. The concentrated extract was filtered through a 0.22 µm filter before analysis.

#### 3.8.2. Determination of the Equilibrium Solubility

The established UHPLC-TQ-MS/MS method was used to determine the equilibrium solubilities of samples.

The equilibrium solubilities of the samples were determined by the shake-flask method [50]. Briefly, the accurately weighed solid sample was carefully added to 10 mL of the aqueous buffer in a glass vial and stirred at (37 ± 1) °C for 24 h. The aliquots of supernatant were taken out and diluted with solvent if necessary, and the concentration of sample in each aliquot was measured by UHPLC-TQ-MS. The injection volume was 2~50 µL, respectively.

## 4. Conclusions

The paper proved that the physical properties—including the surface morphology, particle size, PDI, ZP, specific surface area, porosity, and viscosity—were changed after processing, in which case the bioavailability of BAs in frankincense was improved. The results showed that SEM images of processed frankincense exhibited folds full of holes with a rough surface. Vinegar processing mainly contributes to the crushing process of frankincense, thereby resulting in a decrease of the particle size and an increase of the specific surface area. In addition, the absolute values of the ZP and porosity increased, while the values of the PDI and flow time of simulated gastrointestinal fluid decreased for processed frankincense. Thus, the rates of absorption increased after processing, and processed frankincense provided improved dissolution at different pH values in comparison to frankincense. This study is designed to define and understand the mechanism for the enhancement of bioavailability after vinegar processing, which broadens the horizon of the mechanism of the investigation of processing for TCM.

## Figures and Tables

**Table 1:** RT, related MS data of the six BAs.

No.	Compound	RT (min)	[M + H]^+^ (*m*/*z*)	Daughter Ion (*m*/*z*)	Fragmentor (V)	CE (eV)
Time segments: 0–7 min						
1	KBA	5.522	469.4	391.5 * 407.4	160	30
Time segments: 7–17 min						
2	AKBA	9.292	511	255.4 *	115	11
3	α-BA	10.372	455	437.5 * 372.4	250	37
4	β-BA	15.589	455	377.4 * 437.4	110	35
Time segments: 17–30 min						
5	α-ABA	25.6	497	437.5 * 361.1	120	12
6	β-ABA	27.598	497	437.5 *	120	13

* Quantitative ions.

**Table 2:** Calibration curves, linear ranges, LOD, and LLOQ of six BAs.

No.	Compounds	Regression Equation	r	Linear Range (mg/mL)	LLOQ (mg/mL)	LOD (mg/mL)
1	KBA	*Y* = 2145.1*X* − 25.865	0.9960	0.104~1.04	0.054	0.021
2	AKBA	*Y* = 19549*X* − 151.53	0.9925	0.102~1.02	0.039	0.014
3	α-BA	*Y* = 1169.3*X* − 7.466	0.9997	0.106~1.06	0.087	0.032
4	β-BA	*Y* = 170852*X* + 4894.1	0.9957	0.101~1.01	0.035	0.011
5	α-ABA	*Y* = 300.64*X* − 46.221	0.9987	0.98~9.8	0.79	0.33
6	β-ABA	*Y* = 98.805*X* − 29.213	0.9986	0.98~9.8	0.59	0.28

**Table 3:** Precision and accuracy of six BAs.

Compounds		Precision (RSD%)		Accuracy (CV%)	
	Concentration Levels	Standards Mixture		Extraction of Frankincense	QC Samples
		Intra-Day (n = 6)	Inter-Day (n = 6)		
KBA	L	2.96	2.67	2.46	3.04
	M	1.24	3.21	2.11	0.64
	H	2.46	4.03	1.68	1.05
AKBA	L	5.01	4.96	3.77	2.13
	M	3.84	4.18	3.26	0.78
	H	1.99	1.05	2.55	0.89
α-BA	L	2.80	2.54	0.59	4.66
	M	2.47	3.22	1.21	2.40
	H	4.90	2.01	0.97	2.85
β-BA	L	1.09	3.49	1.91	4.13
	M	3.56	4.54	0.68	3.56
	H	1.43	2.08	2.34	1.19
α-ABA	L	3.88	1.52	3.85	2.34
	M	2.54	2.73	2.44	2.90
	H	4.13	3.05	1.23	3.55
β-ABA	L	3.29	4.00	2.70	3.30
	M	1.17	2.27	2.79	4.01
	H	0.92	2.02	3.43	2.72

**Table 4:** Recovery of six BAs.

	Extraction Samples						QC Samples		
Compounds	Original Amounts (g)	Spiked Amounts (mg)	Detected Amounts (mg)	Recovery (%)	Mean Recovery (%)	RSD (%)	QC Levels	Mean Recovery (%)	RSD (%)
KBA	0.04918	0.750	1.521	102.60	98.44	4.78	L	104.75	1.93
	0.04726	0.750	1.423	93.45					
	0.05012	0.750	1.556	105.36			M	101.63	2.21
	0.05035	0.750	1.498	97.15					
	0.04509	0.750	1.395	94.14			H	104.38	1.05
	0.04728	0.750	1.457	97.94					
AKBA	0.04918	1.235	2.511	105.76	101.01	4.26	L	97.67	3.76
	0.04726	1.235	1.376	98.63					
	0.05012	1.235	2.492	102.35			M	103.40	4.09
	0.05035	1.235	2.390	93.64					
	0.04509	1.235	2.385	103.67			H	96.71	1.98
	0.04728	1.235	2.418	102.00					
α-BA	0.04918	0.920	1.807	97.95	99.12	1.73	L	101.24	2.64
	0.04726	0.920	1.772	97.99					
	0.05012	0.920	1.841	99.76			M	91.68	3.01
	0.05035	0.920	1.820	97.02					
	0.04509	0.920	1.762	101.24			H	105.11	2.47
	0.04728	0.920	1.798	100.77					
β-BA	0.04918	2.40	4.729	99.44	97.97	3.13	L	102.90	4.48
	0.04726	2.40	4.598	97.79					
	0.05012	2.40	4.869	103.41			M	99.53	1.55
	0.05035	2.40	4.704	96.08					
	0.04509	2.40	4.436	95.35			H	106.39	2.20
	0.04728	2.40	4.550	102.60					
α-ABA	0.04918	0.318	0.607	92.54	97.88	4.86	L	102.35	3.14
	0.04726	0.318	0.635	105.18					
	0.05012	0.318	0.641	101.35			M	98.17	1.58
	0.05035	0.318	0.621	94.60					
	0.04509	0.318	0.589	95.05			H	101.04	4.22
	0.04728	0.318	0.614	98.54					
β-ABA	0.04918	2.10	4.080	95.62	96.71	3.72	L	103.82	3.23
	0.04726	2.10	3.891	90.47					
	0.05012	2.10	4.135	96.35			M	106.44	1.79
	0.05035	2.10	4.202	99.08					
	0.04509	2.10	3.956	97.92			H	100.01	2.80
	0.04728	2.10	4.11	100.86					

L: LQC levels, M: MQC levels, H: HQC levels.

**Table 5:** Stability of six BAs.

	QC Samples				Extraction Samples
	Storage Condition/Temperature	Storage Condition/Period	QC Levels	RSD%	RSD%
KBA		6 h	L	1.52	2.53
			M	3.40	
			H	3.11	
	4 °C	9 h	L	2.59	
			M	4.37	
			H	4.05	
	−80 °C	12–16 h	L	1.08	
			M	2.76	
			H	4.43	
	4 °C	30 days	L	3.92	
			M	2.44	
			H	1.67	
AKBA	25 °C	6 h	L	3.03	3.46
			M	3.52	
			H	4.56	
	4 °C	9 h	L	1.91	
			M	2.33	
			H	2.53	
	−80 °C	12–16 h	L	4.78	
			M	3.42	
			H	2.03	
	4 °C	30 days	L	2.72	
			M	3.30	
			H	4.17	
α-BA	25 °C	6 h	L	3.00	2.72
			M	1.35	
			H	4.02	
	4 °C	9 h	L	2.31	
			M	4.28	
			H	3.96	
	−80 °C	12–16 h	L	4.39	
			M	2.88	
			H	2.05	
	4 °C	30 days	L	1.22	
			M	3.64	
			H	3.35	
β-BA	25 °C	6 h	L	2.92	4.39
			M	1.69	
			H	4.87	
	4 °C	9 h	L	1.32	
			M	3.77	
			H	2.33	
	−80 °C	12–16 h	L	2.10	
			M	3.81	
			H	1.54	
	4 °C	30 days	L	4.00	
			M	1.79	
			H	2.85	
α-ABA	25 °C	6 h	L	2.66	2.10
			M	4.23	
			H	2.05	
	4 °C	9 h	L	3.48	
			M	1.77	
			H	2.91	
	−80 °C	12–16 h	L	3.08	
			M	4.33	
			H	1.59	
	4 °C	30 days	L	2.70	
			M	4.40	
			H	3.81	
β-ABA	25 °C	6 h	L	4.74	4.21
			M	2.90	
			H	3.21	
	4 °C	9 h	L	1.68	
			M	2.03	
			H	2.47	
	−80 °C	12–16 h	L	3.88	
			M	1.96	
			H	4.07	
	4 °C	30 days	L	2.85	
			M	2.72	
			H	1.29	

L: LQC levels, M: MQC levels, H: HQC levels.

## References

[1] Frank A., Unger M. (2006). Analysis of frankincense from various Boswellia species with inhibitory activity on human drug metabolising cytochrome P450 enzymes using liquid chromatography mass spectrometry after automated on-line extraction. J. Chromatogr. A.

[2] Mathe C., Culioli G., Archier P., Vieillescazes C. (2004). High-Performance Liquid Chromatographic Analysis of Triterpenoids in Commercial Frankincense. Chromatographia.

[3] Boden S.E., Schweizer S., Ammon H.P.T., Safayhi H. (2000). Concentration-Dependent Potentiating and Inhibitory Effects of Boswellia Extracts on 5-Lipoxygenase Product Formation inStimulated PMNL. Planta Med..

[4] Notarnicola A., Tafuri S., Fusaro L., Moretti L., Pesce V., Moretti B. (2011). The “MESACA” study: Methylsulfonylmethane and boswellic acids in the treatment of gonarthrosis. Adv. Ther..

[5] Sengupta K., Alluri K.V., Satish A.R., Mishra S., Golakoti T., Sarma K.V., Dey D., Raychaudhuri S.P. (2008). A double blind, randomized, placebo controlled study of the efficacy and safety of 5-Loxin® for treatment of osteoarthritis of the knee. Arthritis Res. Ther..

[6] Hamm S., Bleton J., Connan J., Tchapla A. (2005). A chemical investigation by headspace SPME and GC–MS of volatile and semi-volatile terpenes in various olibanum samples. Phytochemistry.

[7] Zhang C., Sun L., Tian R.-T., Jin H.-Y., Ma S.-C., Gu B.-R. (2015). Combination of quantitative analysis and chemometric analysis for the quality evaluation of three different frankincenses by ultra high performance liquid chromatography and quadrupole time of flight mass spectrometry. J. Sep. Sci..

[8] Sharma R., Singh G., Khajuria A., Sidiq T., Singh S., Chashoo G., Pagoch S., Kaul A., Saxena A., Johri R. (2009). In vivo genotoxicity evaluation of a plant based antiarthritic and anticancer therapeutic agent Boswelic acids in rodents. Phytomedicine.

[9] Du Z., Liu Z., Ning Z., Liu Y., Song Z., Wang C., Lu A. (2015). Prospects of Boswellic Acids as Potential Pharmaceutics. Planta Med..

[10] Gerbeth K., Hüsch J., Fricker G., Werz O., Schubert-Zsilavecz M., Abdel-Tawab M. (2013). In vitro metabolism, permeation, and brain availability of six major boswellic acids from Boswellia serrata gum resins. Fitoterapia.

[11] Büchele B., Simmet T. (2003). Analysis of 12 different pentacyclic triterpenic acids from frankincense in human plasma by high-performance liquid chromatography and photodiode array detection. J. Chromatogr. B.

[12] Pan Y.-N., Liang X.-X., Niu L.-Y., Wang Y.-N., Tong X., Hua H.-M., Zheng J., Meng D.-Y., Liu X.-Q. (2015). Comparative studies of pharmacokinetics and anticoagulatory effect in rats after oral administration of Frankincense and its processed products. J. Ethnopharmacol..

[13] Chen L.-L., Verpoorte R., Yen H.-R., Peng W.-H., Cheng Y.-C., Chao J., Pao L.-H., Dai Y. (2018). Effects of processing adjuvants on traditional Chinese herbs. J. Food Drug Anal..

[14] Zhao G.-H., Yan C.-P., Xu Z.-S., Gao Q.-Q., Chen Z.-P., Li W.-D. (2016). The Effect of Salt-Processed Psoralea corylifolia on Generative Organ Targeting. J. Anal. Methods Chem..

[15] Cui X., Qian X., Huang P. (2015). Simultaneous determination of ten flavonoids of crude and wine-processed Radix Scutellariae aqueous extracts in rat plasma by UPLC-ESI-MS/MS and its application to a comparative pharmacokinetic study. Biomed. Chromatogr..

[16] Shilpi S., Sanjula B., Mushir A., Anil K., Javed A. (2009). Solid Dispersion: An Alternative Technique for Bioavailability Enhancement of Poorly Soluble Drugs. J. Dispers. Sci. Technol..

[17] Shekhawat P.B., Pokharkar V.B. (2017). Understanding peroral absorption: Regulatory aspects and contemporary approaches to tackling solubility and permeability hurdles. Acta Pharm. Sin. B.

[18] Göke K., Bunjes H. (2018). Parameters influencing the course of passive drug loading into lipid nanoemulsions. Eur. J. Pharm. Biopharm..

[19] Göke K., Lorenz T., Repanas A., Schneider F., Steiner D., Baumann K., Bunjes H., Dietzel A., Finke J.H., Glasmacher B. (2018). Novel strategies for the formulation and processing of poorly water-soluble drugs. Eur. J. Pharm. Biopharm..

[20] Kollipara S., Gandhi R.K. (2014). Pharmacokinetic aspects and in vitro–in vivo correlation potential for lipid-based formulations. Acta Pharm. Sin. B.

[21] Zhang X., Xing H., Zhao Y., Ma Z. (2018). Pharmaceutical Dispersion Techniques for Dissolution and Bioavailability Enhancement of Poorly Water-Soluble Drugs. Pharmaceutics.

[22] Carrier R.L., Miller L.A., Ahmed I. (2007). The utility of cyclodextrins for enhancing oral bioavailability. J. Control Release.

[23] Curatolo W. (1998). Physical chemical properties of oral drug candidates in the discovery and exploratory development settings. Pharm. Sci. Technol. Today.

[24] Aguiar G.P.S., Marcon M., Mocelin R., Herrmann A.P., Chaves L.M., Piato A.L., Lanza M., Oliveira J. (2017). Micronization of N -acetylcysteine by supercritical fluid: Evaluation of in vitro and in vivo biological activity. J. Supercrit. Fluids.

[25] Dou Z., Li K., Wang P., Cao L. (2012). Effect of Wine and Vinegar Processing of Rhizoma Corydalis on the Tissue Distribution of Tetrahydropalmatine, Protopine and Dehydrocorydaline in Rats. Molecules.

[26] Lu J., Liu L., Zhu X., Wu L., Chen Z., Xu Z., Li W. (2018). Evaluation of the Absorption Behavior of Main Component Compounds of Salt-Fried Herb Ingredients in Qing′e Pills by Using Caco-2 Cell Model. Molecules.

[27] Wu H., Waldbauer K., Tang L., Xie L., McKinnon R., Zehl M., Yang H., Xu H., Kopp B. (2014). Influence of Vinegar and Wine Processing on the Alkaloid Content and Composition of the Traditional Chinese Medicine Corydalis Rhizoma (Yanhusuo). Molecules.

[28] Aretz A., Ehle L., Haeusler A., Bobzin K., Öte M., Wiesner S., Schmidt A., Gillner A., Poprawe R., Mayer J. (2018). In situ investigation of production processes in a large chamber scanning electron microscope. Ultramicroscopy.

[29] Sun C., Muller E., Meffert M., Gerthsen D. (2018). On the Progress of Scanning Transmission Electron Microscopy (STEM) Imaging in a Scanning Electron Microscope. Microsc. Microanal..

[30] Bansal M., Mittal N., Yadav S.K. (2018). Periodontal thermoresponsive, mucoadhesive dual antimicrobial loaded in-situ gel for the treatment of periodontal disease: Preparation, in-vitro characterization and antimicrobial study. J. Oral. Biol. Craniofac. Res..

[31] Chinese Pharmacopoeia Commission (2015). Pharmacopoeia of People’s Republic of China.

[32] Li Q., Chen F., Liu Y., Yu S., Gai X., Ye M., Yang X., Pan W. (2018). A novel albumin wrapped nanosuspension of meloxicam to improve inflammation-targeting effects. Int. J. Nanomed..

[33] Badie H., Abbas H. (2018). Novel Small Self-Assembled Resveratrol -Bearing Cubosomes and Hexosomes: Preparation, Charachterization and Ex Vivo Permeation. Drug Dev. Ind. Pharm..

[34] Ikonen M., Murtomäki L., Kontturi K. (2010). Microcalorimetric and zeta potential study on binding of drugs on liposomes. Colloids Surf. B Biointerfaces.

[35] Chen L., Wang Y., Zhang J., Hao L., Guo H., Lou H., Zhang D. (2014). Bexarotene nanocrystal—Oral and parenteral formulation development, characterization and pharmacokinetic evaluation. Eur. J. Pharm. Biopharm..

[36] Gigliobianco M.R., Casadidio C., Censi R., Di Martino P. (2018). Nanocrystals of Poorly Soluble Drugs: Drug Bioavailability and Physicochemical Stability. Pharmaceutics.

[37] Sebe I., Szabo P., Kállai-Szabó B., Zelkó R. (2015). Incorporating small molecules or biologics into nanofibers for optimized drug release: A review. Int. J. Pharm..

[38] Kim H.-I., Park S.Y., Park S.J., Lee J., Cho K.H., Jee J.-P., Kim H.-C., Maeng H.-J., Jang D.-J. (2018). Development and Evaluation of a Reconstitutable Dry Suspension to Improve the Dissolution and Oral Absorption of Poorly Water-Soluble Celecoxib. Pharmaceutics.

[39] Gibson N., Rauscher H., Roebben G. (2016). Comments on the article by A. J. Lecloux (J Nanopart Res (2015) 17:447) regarding the use of volume-specific surface area (VSSA) to classify nanomaterials. J. Nanopart. Res..

[40] Vasvári G., Kalmár J., Veres P., Vecsernyés M., Bácskay I., Fehér P., Ujhelyi Z., Haimhoffer Á., Rusznyák Á., Fenyvesi F. (2018). Matrix systems for oral drug delivery: Formulations and drug release. Drug Discov. Today Technol..

[41] Valmikinathan C.M., DeFroda S., Yu X. (2009). Polycaprolactone and Bovine Serum Albumin Based Nanofibers for Controlled Release of Nerve Growth Factor. Biomacromolecules.

[42] Mandal S., Ray R., Basu S.K. (2012). Evaluation of a Matrix Tablet Prepared with Polyacrylamide-g-Sodium Alginate Co-polymers and Their Partially Hydrolyzed Co-polymers for Sustained Release of Diltiazem Hydrochloride. J. Biomater. Sci. Polym. Ed..

[43] Sjögren E., Westergren J., Grant I., Hanisch G., Lindfors L., Lennernäs H., Abrahamsson B., Tannergren C. (2013). In silico predictions of gastrointestinal drug absorption in pharmaceutical product development: Application of the mechanistic absorption model GI-Sim. Eur. J. Pharm. Sci..

[44] Xue B., Zhao Y., Su J. (2017). In Vitro Intestinal Absorption and Metabolism of Magnoflorine and its Potential Interaction in Coptidis Rhizoma Decoction in Rat. Eur. J. Drug Metab. Pharmacokinet..

[45] Wilson T.H., Wiseman G. (1954). The use of sacs of everted small intestine for the study of the transference of substances from the mucosal to the serosal surface. J. Physiol..

[46] Barthe L., Woodley J., Houin G. (1999). Gastrointestinal absorption of drugs: Methods and studies. Fundam. Clin. Pharmacol..

[47] Perrier J., Zhou Z., Dunn C., Khadra I., Wilson C.G., Halbert G. (2018). Statistical investigation of the full concentration range of fasted and fed simulated intestinal fluid on the equilibrium solubility of oral drugs. Eur. J. Pharm. Sci..

[48] Baka E., Comer J.E., Takács-Novák K. (2008). Study of equilibrium solubility measurement by saturation shake-flask method using hydrochlorothiazide as model compound. J. Pharm. Biomed. Anal..

[49] Ning Z., Wang C., Liu Y., Song Z., Ma X., Liang D., Liu Z., Lu A. (2018). Integrating Strategies of Herbal Metabolomics, Network Pharmacology, and Experiment Validation to Investigate Frankincense Processing Effects. Front. Pharmacol..

[50] Guo N., Yang D., Ablajan K., Niu X., Fan B., Wang Z., Dai J., Wu X., Liu B. (2013). Simultaneous quantitation of seven alkaloids in processed Fuzi decoction by rapid resolution liquid chromatography coupled with tandem mass spectrometry. J. Sep. Sci..

